# Calculation of the Respiratory Modulation of the Photoplethysmogram (DPOP) Incorporating a Correction for Low Perfusion

**DOI:** 10.1155/2014/980149

**Published:** 2014-08-07

**Authors:** Paul S. Addison, Rui Wang, Scott J. McGonigle, Alberto A. Uribe, Sergio D. Bergese

**Affiliations:** ^1^Covidien Respiratory & Monitoring Solutions, Edinburgh EH26 0PJ, UK; ^2^Department of Anesthesiology, The Ohio State University Wexner Medical Center, Columbus, OH 43210, USA; ^3^Department of Neurological Surgery, The Ohio State University Wexner Medical Center, Columbus, OH 43210, USA

## Abstract

DPOP quantifies respiratory modulations in the photoplethysmogram. It has been proposed as a noninvasive surrogate for pulse pressure variation (PPV) used in the prediction of the response to volume expansion in hypovolemic patients. The correlation between DPOP and PPV may degrade due to low perfusion effects. We implemented an automated DPOP algorithm with an optional correction for low perfusion. These two algorithm variants (DPOPa and DPOPb) were tested on data from 20 mechanically ventilated OR patients split into a benign “stable region” subset and a whole record “global set.” Strong correlation was found between DPOP and PPV for both algorithms when applied to the stable data set: *R* = 0.83/0.85 for DPOPa/DPOPb. However, a marked improvement was found when applying the low perfusion correction to the global data set: *R* = 0.47/0.73 for DPOPa/DPOPb. Sensitivities, Specificities, and AUCs were 0.86, 0.70, and 0.88 for DPOPa/stable region; 0.89, 0.82, and 0.92 for DPOPb/stable region; 0.81, 0.61, and 0.73 for DPOPa/global region; 0.83, 0.76, and 0.86 for DPOPb/global region. An improvement was found in all results across both data sets when using the DPOPb algorithm. Further, DPOPb showed marked improvements, both in terms of its values, and correlation with PPV, for signals exhibiting low percent modulations.

## 1. Introduction

DPOP (Delta-POP or ΔPOP) is a physiological parameter calculated from the pulse oximeter signal—the photoplethysmogram or “pleth”—which measures the strength of respiratory modulations present in the waveform. The parameter has been shown to be useful as an index of fluid responsiveness with many studies showing favourable correlation between it and pulse pressure variation (PPV), often used in the determination of the response to volume expansion [1–7]. PPV is, however, an invasive parameter requiring an arterial line, whereas a measure based on the pulse oximeter would provide an entirely noninvasive technology. This is the main driver of the current interest in this area.

Cannesson et al. [8] suggested DPOP as measure of the “respiratory variation in pulse oximetry plethysmographic (POP) waveform amplitude” and defined it as follows:

(1) $$\begin{array}{c} \text{DPOP}=\frac{\left({\text{AMP}}_{\max}-{\text{AMP}}_{\min}\right)}{{\text{AMP}}_{\text{ave}}}, \end{array}$$

where AMP is the amplitude of the cardiac pulse waveforms in the pleth, AMP_ave_ = (AMP_max⁡_ + AMP_min⁡_)/2. DPOP is usually expressed as a percentage. Note that the equation for DPOP has the same mathematical formulation as PPV [9, 10] and other similar formulations for pleth-based fluid responsiveness parameter were proposed by other groups at around the same time as Cannesson's 2005 paper [11, 12].

**Figure 1:**
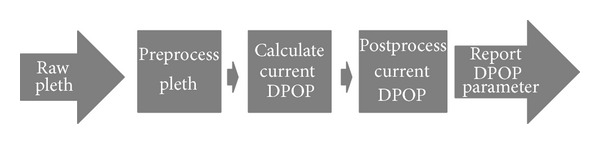
High-level schematic overview of the DPOP algorithm.

The basic computation of DPOP, expressed by (1), is relatively simple, requiring only a few lines of computer code to implement. However, for the development of a fully automated algorithm capable of coping with the extremes of data characteristics in the clinical environment, significantly more processing is required. A fully automated DPOP algorithm can be broken into three main parts: (1) preprocessing, where the raw pleth is manipulated prior to the computation of DPOP; (2) processing, where the computation of the DPOP value according to (1) is carried out; and (3) postprocessing, where the current computed value of DPOP is further processed. This high-level structure of the algorithm is shown in Figure 1. Preprocessing and postprocessing activities dominate all commercial algorithms for physiological parameters. This sophistication is necessary in order to produce a fully automated algorithm with the necessary logic and decision making processes required to provide a robust, clinically useful number for display [13]. The pre- and postprocessing steps involve filtering of the raw pleth waveform, assessment of the quality of the signal coming in, removal of irregular pulse waveforms, identification and removal of outlying data points, smoothing, and, finally, incorporation of the most recently calculated value within the reported value (i.e., where the value calculated over the recent segment of signal is used to update the value displayed to the clinician). This latter step may include an average of a number of previous points weighted by temporal relevance and the quality of the data.

We coded a DPOP algorithm incorporating the steps described above. During the development of the DPOP code we found that an improvement in the correlation between DPOP and PPV could be achieved at low perfusion values by adding an extra postprocessing code module. Problems with low perfusion are well documented in the literature and, in fact, low perfusion is cited as an exclusion criterion in the studies by Cannesson [1, 2] and Chandler et al. [7]. Hence, we developed two versions of the algorithm: DPOPa, which provides a robust DPOP parameter as given by the formulation of (1) and DPOPb, which modifies the DPOP value at perfusion indices <3% by decreasing it proportionately as the perfusion index tends to zero.

The aim of the present study reported here was to assess the relative performance of the two photoplethysmographic-derived indices in terms of correlation and agreement against the pulse pressure-derived PPV measure in patients in the operating room undergoing controlled mechanical ventilation.

## 2. Methods

### 2.1. Patients

With institutional review board approval and written informed consent, a convenience sample of adult patients was enrolled at the Ohio State University (OSU) Wexner Medical Center (Principal Investigator: Sergio D. Bergese, MD). Ventilated patients requiring placement of an intra-arterial line who have been scheduled to undergo elective surgery were enrolled in the study. No specific disease states or pathophysiologic conditions were targeted during enrolment. Exclusion criteria were (1) currently participating in or having participated in an investigational drug study within seven days of enrolment, (2) having known severe contact allergies to adhesive materials, (3) having existing health conditions preventing proper sensor application, and (4) belonging to vulnerable groups (pregnant, breastfeeding, and prisoners).

### 2.2. Data Acquisition

Each patient was fitted with a finger sensor (Nellcor OxiMax Max-A, Covidien, Boulder CO) as per the sensor's device labelling. The sensor was connected to a custom data-recording box that contained a Nellcor OEM pulse oximeter of the same type found in the commercially available N-600x monitor (Nell-1 board, Covidien, Boulder CO). High resolution pleth waveforms were collected from the Nell-1 board which was configured in its default, “Normal,” response mode. There were no displayed readings or alarms with this investigational recording system; the hospital's bedside or multiparameter pulse oximeter was used for patient care. The blood pressure signal from an intra-arterial blood pressure monitor (Solar 8000, by GE-Marquette) was also recorded. This passed through an Ethernet cable to a laptop with BedMaster Software (Exel Medical, Florida). A synchronized acquisition of the pulse oximeter and arterial pressure signals was performed during the whole procedure and saved to a laptop for later analysis.

Demographic information, sensor types and sensor sites used, and intraoperative data sheets for manual listing of significant events during the data acquisition were collected as useful references for informing the data analysis. These events included the administration of drugs (both time and type of drug), the times of significant patient motion, surgical incisions, washing patients, attachment to a heart lung machine, time of significant changes (±20 mm Hg) of blood pressure, induction, extubation, central line insertion, patient/patient bed position change, arterial line rezeroing, flushing, or height adjustments.

### 2.3. Data Analysis

The resulting OR data set comprised 36 patient records where pleth and arterial line waveforms were collected simultaneously. 16 data sets were excluded from analysis for a variety of reasons, including the absence of, or missing, information in the case report form (CRF), absence of a blood pressure waveform recording, absence of a pleth waveform recording, presence of an arrhythmia, corrupted data files, and pleth data with artifacts due to BP cuff inflations on the same arm as the oximeter probe. The remaining 20 subjects had a mean length of data record of 115 minutes, with the shortest recording of 43 minutes and longest recording 204 minutes. All subjects were provided with general anaesthetic drugs before and during the procedure. 14 subjects were also provided with vasoactive drugs at specific points during the procedure.

**Figure 2:**
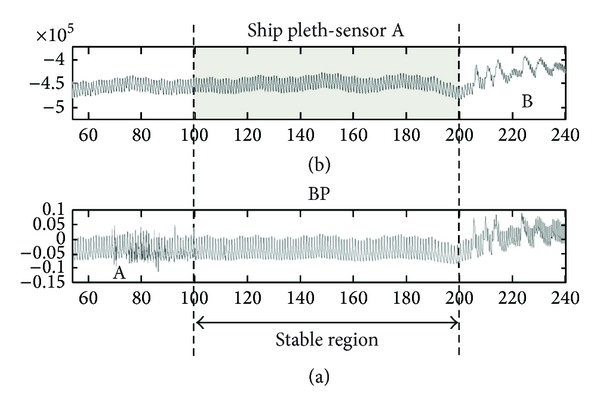
Selection of a stable region ((b) finger pleth and (a) arterial BP).

The collected signals were subdivided into two distinct data sets: (1) a stable region data set and (2) a global data set. The “stable data set” corresponds to a few minutes of high quality signal segments selected from within the post-inducation, pre-incision period, where only general anaesthetic drugs had been administered (i.e., no vasoactive drugs) and where the pleth and BP signal were reasonably artifact-free. The stable data set is intended to provide a comparison to results from studies based on manually selected high signal quality regions often reported in the literature. The identification of the stable region was performed by visual inspection. An example of a stable region selected for analysis is shown in Figure 2. The artifact in the BP signal within region A caused the exclusion of this part of both signals for analysis (although the pleth signal is of relatively good quality here). The artifact in both signals within region B caused the exclusion of this part of the signals from the stable region data set. The selected pleth region is shown shaded in the plot. The “global data set” contains the entire data record of the patient (i.e., including sections such as A and B in Figure 2) and is indicative of all the data encountered by a commercial device in practice. The global data set provides a much tougher test for an automated algorithm which has to make decisions on signal quality and optimize the reported parameter accordingly via advanced signal processing measures.

### 2.4. Statistical Analysis

The performance analysis of the DPOP parameter against the PPV signal comprises computing statistics that describe quantitatively the relationship between them, including correlation and receiver operator characteristic (ROC) curve analysis. A best fit line for DPOP versus PPV was plotted based on linear least square regression. The Pearson correlation coefficient, *R*, describes how well DPOP fits the linear relationship with PPV. The statistical significance (*P* value) of *R* was also calculated. By computing a ROC curve, we also determined sensitivity, specificity, and area under the curve (AUC) values. These correspond to the hypothetical substitution of DPOP for PPV achieved by setting a fixed threshold for PPV of 13%, as used in several studies for indicating the boundary between nonresponsive and responsive patients (e.g., de Figueiredo et al. [11], Cannesson et al. [8], Natalini et al. [12], Landsverk et al. [14], and Westphal et al. [5]). A ROC curve is then computed by determining the sensitivity and specificity pairs over a range of DPOP thresholds. We selected the optimal DPOP threshold from the predefined criterion of maximising the Youden index (sensitivity + (specificity − 1)) [6, 12, 15].

## 3. Results

**Figure 3:**
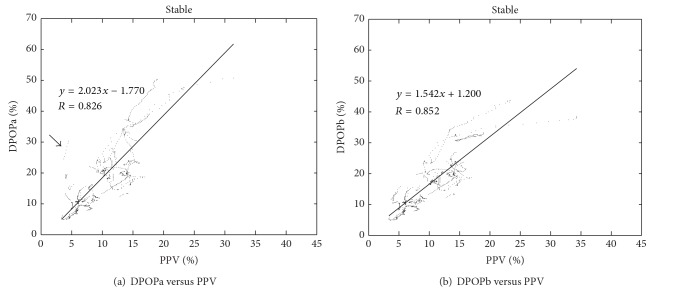
Original and Percent modulation (PMod) corrected DPOP results for stable regions. Note: vertical axes are set to the same scale across plots.

The results of the analysis are summarized in Table 1.  Figure 3(a) shows a plot of DPOPa versus PPV for the stable regions of the OSU data set, corresponding to an *R* value of 0.83.  Figure 3(b) shows the corresponding plot for DPOPb for the stable data set which can be compared to the DPOPa results in Figure 3(a). The correction for low perfusion causes a small improvement of the *R* number for the stable region runs (from 0.83 to 0.85). Although the overall correlation improves very slightly, it is obvious when comparing the plots of Figures 3(a) and 3(b) that a distinct subset of outlier data points (indicated by the arrow in Figure 3(a)) is dealt with by DPOPb algorithm. The removal of outliers in this way is a critically important part of algorithm design, which should not be driven solely by the improvement in global statistics (e.g., *R*, AUC, sensitivity, specificity, etc.). In fact, outliers often provide the largest hurdle in the design of clinical algorithms.

**Figure 4:**
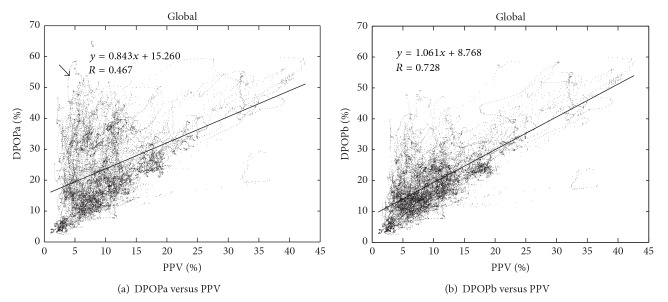
Original and PMod corrected DPOP results for global regions. Note: vertical axes are set to the same scale across plots.

Figure 4(a) shows the scatter plot of DPOP versus PPV for the global data records of the OSU data set. This plot clearly exhibits poorer performance than the corresponding stable region plot of Figure 3(a), with a lower value of *R* of 0.47.  Figure 4(b) shows the corresponding plot for DPOPb. However, for this data set we see an improvement in the global statistics due to the correction for low perfusions, with *R* increasing from 0.47 to 0.73. An arrow marks the region in Figure 4(a) plot for the DPOPa data where many points exhibit distinctly greater values than expected for the corresponding PPV values. We can see from Figure 4(b) that many of these outlier points are removed from this region by the low perfusion correction, resulting in the pronounced increase in correlation between DPOP and PPV.

**Figure 5:**
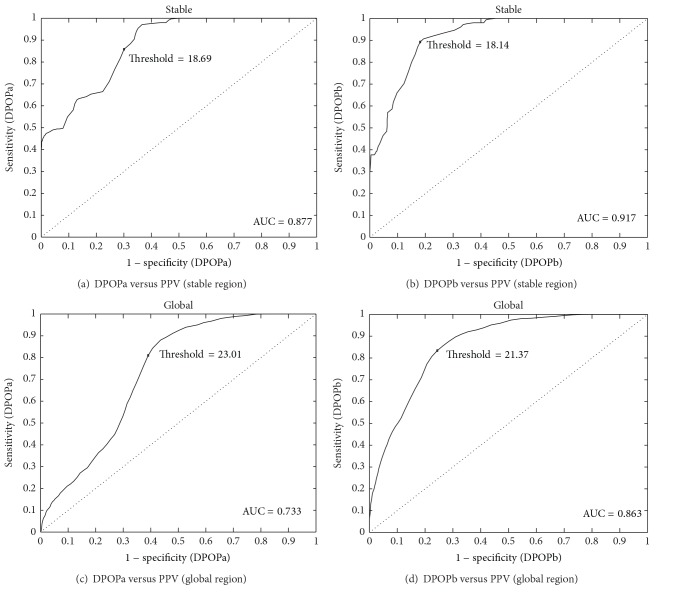
ROC curves corresponding to the data in Figures 3 and 4.

Figure 5 contains the ROC curves corresponding to the results data plotted in Figures 3 and 4. The points corresponding to the maximum Youden index are superimposed on the curves for reference. The ROC statistics are summarized in Table 1, including the sensitivity and specificity at the maximum Youden index and the DPOP threshold that this operating point corresponds to. The stable region data has relatively high ROC statistics: sensitivities of 0.86 and 0.89, specificities of 0.70 and 0.82, and AUCs of 0.88 and 0.92 for DPOPa and DPOPb, respectively. The corresponding values for the global data set are sensitivities of 0.81 and 0.83, specificities of 0.61 and 0.76, and AUCs of 0.73 and 0.86, respectively, highlighting both the poorer results due to distinctly noisier characteristics of the global data and the ability of the DPOPb algorithm to improve results in both cases.

## 4. Discussion and Conclusion

The present study demonstrates a strong relationship between DPOP and PPV and its further improvement through accounting for low perfusion signals within the algorithm. In addition, DPOP was able to identify PPV values either side of a 13% threshold with high sensitivity and specificity.

DPOP has been proposed as a noninvasive surrogate for pulse pressure variation (PPV) used in the determination of the response to volume expansion in hypovolemic patients [8, 11] and many studies have found good agreement between the two parameters in the OR and ICU [1–8, 11, 12]. Although considered a minimally invasive technique, the placement of a peripheral arterial line is time-consuming and associated on rare occasion with potentially harmful complications, including infection, thrombosis, and hematoma [5]. Numerous studies have now reported on the DPOP parameter and many research groups have constructed algorithms for its computation from first principles where the implementation details have been described (e.g., Cannesson et al. [1–3], Feissel et al. [4], Westphal et al. [5], Høiseth et al. [6], and Chandler et al. [7]). However, a number of authors draw attention to the already preprocessed nature of the pleth with which they are working. This is aptly summed up by Cannesson et al. [16] who state that it is well known that the pleth is “a highly processed signal, and that only the raw waveform can display consistent respiratory variations.” Landsverk et al. [14] echo the sentiment commenting that the commercial pulse oximeter used in their study had “filters built in” and hence the analog output signal they used was therefore not a “raw signal.” They therefore could not exclude the possibility that the respiratory variations could have been altered by the preprocessing of the device. Cannesson et al. [3] state that visual analysis of the respiratory variations in the waveform is unreliable, since the amplitude of the curve “is constantly processed and smoothed by most of the devices commercially available.” Delerme et al. [17] describe the plethysmographic signal as a “highly processed and position dependent signal” and results might vary from one pulse oximeter to another. In the present study, we are fortunate in our ability to access the raw pleth waveform and manipulate it in order to mitigate both poor quality signal and low perfusion effects. In addition, we have access to a toolbox of tried and tested signal processing modules specific to the extraction of respiratory modulations from the pleth on which to base the algorithm [13, 18].

There is a temptation to believe the pulse pressure waveform and pulsatile pleth waveform to be essentially the same signal, and indeed Westphal et al. [5] comment on the resemblance between the two. However, additional complex, nonlinear pressure-mechanical coupling and light absorption phenomena separate the physiological system that provides us with the pleth from that of the arterial waveform. Thus a correction is required for DPOP at low perfusions as the pressure-mechanical dynamical system changes state. The correction we employed for low perfusion (DPOPb) improved all results in the present study. The most marked improvement was for the more challenging global data set where *R* improved from 0.47 to 0.73. Detailed visual inspection of the pleth waveforms indicates that, as perfusion lowers, there appears to be a differentially faster decrease in the mean component of the DPOP calculation (the denominator of (1)) than in the modulation component (the numerator). This behaviour should not be surprising due to the complex nonlinear nature of the pressure-mechanical interaction of the pulse wave at the vessel wall [19, 20]. In addition, studies have shown that the DPOP and PPV parameters vary differently during the hypovolemic state due to the changing relationship between the stroke volume and pulse pressure when the compliance of the aorta is greatly increased [21, 22]. Thus we see from both physiomechanical and phenomenological considerations that there is an expectation of deviation from a linear relationship. (It is also the reason we should not expect a 1 : 1 linear relationship between DPOP and PPV.) Many studies have, in fact, commented on the deleterious effect of variable and/or low perfusion levels on methods to extract respiratory modulation information from the pleth [6, 8, 23, 24] and some, in fact, cite low perfusion as a criterion for excluding the data from analysis [1, 2, 7]. Furthermore, Broch et al. [25] clearly demonstrated the dependency of a commercially available pleth-based respiratory modulation strength parameter (PVI) on perfusion index. They stated that “PVI showed a poor ability to predict fluid responsiveness if different perfusion states of the index finger were not considered” and concluded that the parameter had limited capabilities to predict fluid responsiveness in the presence of low perfusion.

**Figure 6:**
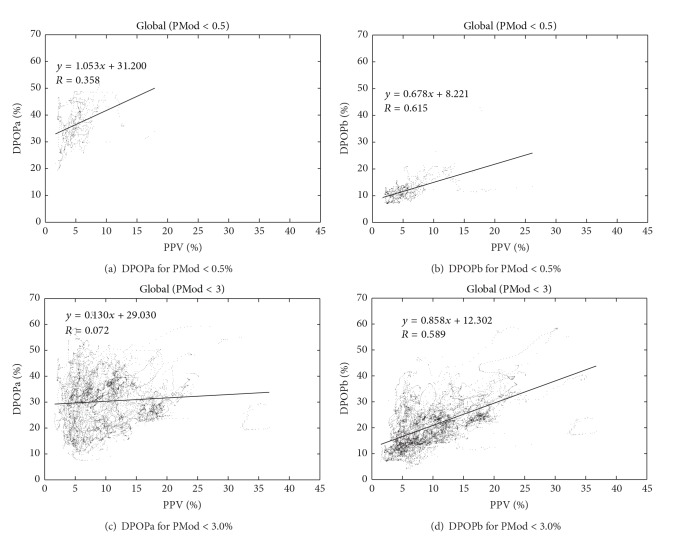
DPOP results for low PMod values.

To further investigate the performance of the algorithm at low perfusive states we considered subsets of the data at percent modulations below a range of threshold values. Percent modulation, PMod, is the ratio of the pulse amplitude to the overall signal intensity. We reran the algorithm for a range of PMod thresholds equal to 0.5%, 1%, 2%, and 3%. Some examples of the results are presented in Figure 6. Figures 6(a) and 6(b) contain the global data split into two subgroups corresponding to a PMod less than 0.5%—one corresponding to DPOPa and the other to DPOPb. We can see that, through the correction for low perfusion, the DPOP values are moved down to those more in line with the relationship depicted in Figure 4(b). In addition, the corresponding *R* value improves from 0.36 to 0.62. Figures 6(c) and 6(d) contain similar plots for data with PMod values less than 3%. Again we see both an improvement in the location of the data and a marked improvement in the correlation coefficient, *R* (from 0.07 to 0.60). Similar behaviour was found for the 1% and 2% threshold, which gave improvements in *R* from 0.13 to 0.73 and 0.22 to 0.63, respectively (not shown).

**Figure 7:**
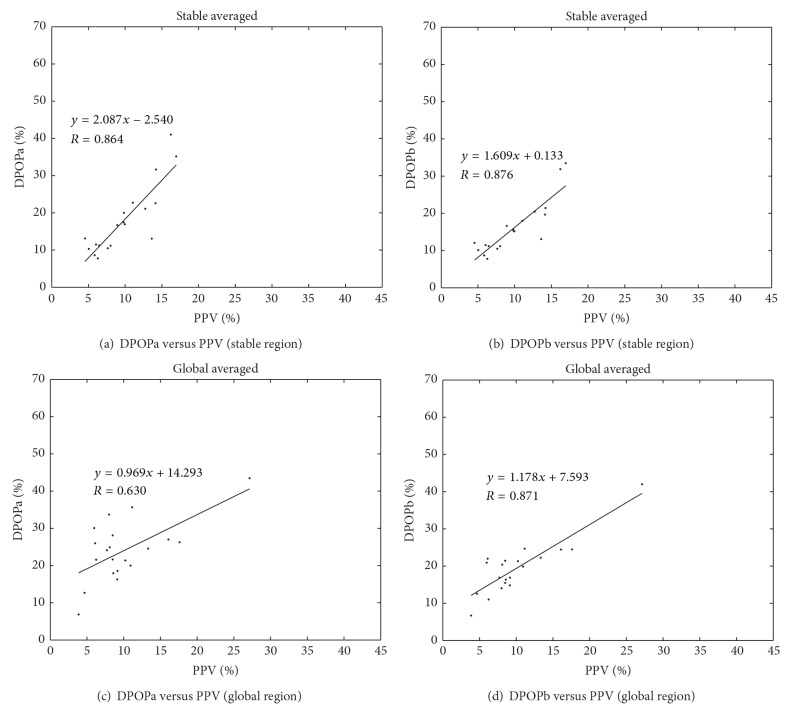
DPOP results reported on a one-point-per-patient basis.

The present results fit well with most previous studies involving OR data which show good correlation between DPOP and PPV, with 5 out of 6 reported studies exhibiting *R* values above 0.7 [2, 3, 6, 8, 15]. The remaining study carried out by de Souza Neto et al. [26] reported *R* = 0.48. Note that Hengy et al. [15] found a relatively high *R* value of 0.79 for the correlation between mean values for all patients and lower value of *R* = 0.47 when considering the mean value of intrapatient coefficients of correlation. Hengy also manually deleted 5.7% of the respiratory cycles because of poor quality signal, whereas, in the present study, the whole data record was fed into the algorithm. (In fact, this is a prime requirement of our automated algorithm: it has to work with all signal characteristics encountered in practice.) The literature contains a wide range of methods for pre- and postprocessing of the data. Many studies have performed manual selection of optimal data segments, while others have attempted to automate the process. This may account for some of the variability in reported values. However, it should also be noted that many studies resort to plotting a single averaged data point per subject, and often this may be averaged over a few hand-picked respiratory cycles, while others attempt much longer term averaging schemes. Averaging can greatly increase the signal to noise ratio of the system and generally leads to improved results. As an example, Figure 7 contains the correlation plots corresponding to the stable and global regions depicted in Figures 3 and 4, but with a single point representing the mean of the data for each subject. The correlations improve for all data sets, with stable region *R* values for DPOPa and DPOPb showing slight improvements to 0.86 and 0.88, respectively. The *R* values for DPOPa and DPOPb in the global region, however, exhibit distinct improvements from *R* = 0.47 to 0.63 and 0.73 to 0.87, respectively, as the global averaging proves particularly good at suppressing the signal noise in this more challenging data set. Thus care has to be taken when interpreting reported results. Plotting in this way may serve some use in that it denoises the data in a global sense, leading to an improved resolution of a distinct underlying relationship. However, it does not represent the reported parameter in practice which is computed over a more localised signal segment and therefore not amenable to the improved signal to noise characteristics provided by very long-term averaging. Reported results should, therefore, be interpreted in the context of the method used to produce them. Cannesson et al. [16] echoes this sentiment arguing that for such studies “the way in which the data are recorded, analyzed, and reported should be standardized in order to avoid potential confounding factors.”

In conclusion, a sophisticated algorithm for the determination of a robust DPOP parameter has been developed which serves as a proxy for PPV information, in terms of its correlation with PPV and its ability to predict PPV values either side of a predefine threshold (of 13%). We further enhanced the performance of the parameter by providing a correction for low perfusion signals.

## Figures and Tables

**Table 1:** Original and PMod corrected DPOP results.

Parameter	Data set	*R*	*P*	Sensitivity	Specificity	DPOP threshold	AUC
DPOPa	Stable	0.826	<0.01	0.858	0.699	18.69	0.877
DPOPb	Stable	0.852	<0.01	0.892	0.819	18.14	0.917
DPOPa	Global	0.467	<0.01	0.810	0.609	23.01	0.733
DPOPb	Global	0.728	<0.01	0.834	0.756	21.37	0.863
